# Choline PET/CT in recurrent prostate cancer

**DOI:** 10.3389/fonc.2023.1079808

**Published:** 2023-03-21

**Authors:** Beatrice Detti, Maria Grazia Carnevale, Sara Lucidi, Luca Burchini, Saverio Caini, Carolina Orsatti, Niccolò Bertini, Manuele Roghi, Vanessa di Cataldo, Simona Fondelli, Gianluca Ingrosso, Giulio Francolini, Daniele Scartoni, Angela Sardaro, Antonio Pisani, Silvia Scoccianti, Cynthia Aristei, Lorenzo Livi

**Affiliations:** ^1^ Radiation Oncology Unit, Azienda Ospedaliero-Universitaria Careggi, Florence, Italy; ^2^ Department of Experimental and Clinical Biomedical Sciences “M. Serio”, University of Florence, Florence, Italy; ^3^ Institute for Cancer Research, Prevention and Clinical Network - Istituto per lo Studio e la Prevenzione Oncologia (ISPRO), Florence, Italy; ^4^ Struttura Organizzativa Complessa (SOC) Radioterapia Oncologica, Ospedale Santa Maria Annunziata, Bagno a Ripoli, Firenze, Azienda Unità Sanitaria Locale (USL) Toscana Centro, Florence, Italy; ^5^ Radiation Oncology, Department of Surgical and Biomedical Science, University of Perugia and Perugia General Hospital, Perugia, Italy; ^6^ Proton Therapy Center-Azienda Provinciale per i Servizi Sanitari, Trento, Italy; ^7^ Radiotherapy Unit, University of Bari, Bari, Italy

**Keywords:** [18F]-choline PET/CT, prostate cancer, biochemical relapse, stereotactic radiotherapy, stereotactic ablative radiotherapy, metastasis-directed therapy

## Abstract

**Purpose:**

Biochemical recurrence (BR) occurs in up to 40% of patients with prostate cancer (PCa) treated with primary radical prostatectomy (RP). Choline PET/CT may show, in a single-step examination, the site of tumor recurrence earlier than traditional imaging methods, particularly at low prostate-specific antigen (PSA) levels, thus influencing subsequent treatment.

**Methods/patients:**

Patients with recurrent and non-metastatic prostate cancer (nmPCa), who were assessed with choline PET/CT, were included in the analysis. Based on imaging results, the following therapeutic strategies were chosen: radiotherapy to the prostatic bed, androgen deprivation therapy (ADT), and chemotherapy or stereotactic body radiotherapy (SBRT) to either the pelvic lymph nodes or distant metastases. We assessed the impact of age, PSA levels, Gleason score (GS), and adjuvant therapy on oncological outcomes.

**Results:**

Data from 410 consecutive nmPCa patients with BR who underwent RP as primary treatment were analyzed. One hundred seventy-six (42.9%) patients had a negative choline PET/CT, and 234 (57.1%) patients resulted positive. In the multivariate analysis, only chemotherapy and PSA at recurrence were significant independent prognostic factors on overall survival (OS). In the PET-positive subgroup, the number of relapses, PSA post-prostatectomy, and chemotherapy impacted on OS. PSA (post-surgery and at recurrence) affected progression-free survival (PFS) in the univariate analysis. In the multivariate analysis, GS, the number of relapse sites, and PSA (post-surgery and at recurrence) were significant prognostic factors for disease-free survival (DFS).

**Conclusion:**

Choline PET/CT provides better accuracy than conventional imaging for the assessment of nmPCa with BR after prostatectomy, thereby enabling salvage strategies and improving quality of life.

## Introduction

Prostate cancer (PCa) still accounts for most tumor cases and is the third leading cause of cancer-related deaths in men worldwide (1). Among standard options for localized PCa, almost half of the patients undergo radical prostatectomy (RP) (2). Up to 40% of cases treated with RP eventually develop biochemical recurrence (BR) (3), and the risk is greater among patients with high-risk features, namely, extraprostatic extension, seminal vesicle invasion, and positive surgical margins (4). Restaging imaging modalities have a pivotal role in identifying any possible site of local and/or metastatic recurrence, thus affecting the therapeutic management. In this setting, salvage modalities are of emerging interest for both local and distant oligo-recurrences with encouraging improvements in the delay of systemic therapy and quality of life (5). Conversely, in case of distant metastases, patients are usually treated with androgen deprivation therapy (ADT), chemotherapy, or radiotherapy according to the burden of disease. A combination of computed tomography (CT), magnetic resonance imaging (MRI), and bone scintigraphy has been the conventional imaging modality (CIM) thus far, despite being limited by poor accuracy, particularly in the oligometastatic setting and at low prostate-specific antigen (PSA) levels (6). This has prompted the advent of whole-body positron emission tomography/computed tomography (PET/CT) with labeled radiotracers. Choline-based PET/CT, both [^11^C]- and [^18^F]-labeled choline derivates, has been the most extensively studied as a promising molecular imaging modality for the evaluation of PCa with BR, proving both higher sensitivity and specificity than CIM in detecting relapse or metastatic spread, in relation to the PSA level and its kinetics (7, 8). Owing to the growing body of literature, recent EAU guidelines recommend choline PET/CT as a diagnostic approach for patients fit for salvage treatments after primary therapy. However, following RP, the use of choline PET/CT has not yet reached a unanimous consensus, and to date, only weak recommendations exist for those cases where results would influence subsequent treatment decisions (9, 10). The heterogeneity of the studies could be one of the reasons that have hindered a correct understanding of the potential role of choline PET/CT imaging in recurrent PCa, the optimal timing, and the clinical impact on the management of secondary therapy. Thus, the aim of the present study is to retrospectively evaluate the potential prognostic role of choline PET/CT, to identify sites of recurrent disease in patients with a rising PSA after primary therapy for prostate cancer.

## Materials and methods

### Study design and characteristics of the study population

We retrospectively analyzed patients with non-metastatic prostatic cancer (nmPCa) referred to our center and to other collaborative centers for recurrent PCa from 2001 to December 2017. The inclusion criteria were RP as the primary treatment, with or without adjuvant radiotherapy or ADT. Patients were stratified into risk classes (low/intermediate and high according to Gleason score, GS) and by PSA at diagnosis (>/≤ median value of 10 ng/ml). BR was defined as two consecutive increases of the PSA value over >0.2 ng/ml. The following clinical parameters were assessed: age, PSA value (basal, after surgery, and at the time of BR), GS, stage, adjuvant therapy, and treatment strategy at BR. The study was performed according to national regulations and to local committee recommendations. All patients gave general permission for the use of their clinical data for scientific purposes and informed consent for the anonymous publication of data.

### Acquisition and analysis of PET/CT

PET-CT was performed on a GE Healthcare Discovery scanner with the patient in conditions of rest. A flat dose of 350 MBq of fluoro-18 ethylcholine (^18^F-choline PET/CT) was injected intravenously. PET images were acquired from the skull base to the pelvis after 60 min. A low-dose, non-contrast CT was also acquired: 140 kV; 80 mA; tube rotation time 0.5 s per rotation; and 3.75 mm sections, thickness corresponding to the PET section. Images were reconstructed using a standard OSEM algorithm; both the non-attenuation-corrected and the attenuation-corrected datasets were reconstructed. The protocol was applied uniformly in all collaborative centers.

### Statistical analysis

The patients’ characteristics were described using medians and interquartile ranges (IQRs) for continuous variables and number and percentages for categorical variables and compared using Wilcoxon’s rank-sum test and Fisher’s exact test, respectively. Distant progression-free survival (DPFS) was defined as evidence of new lesions outside the primary site or last follow-up. Progression-free survival (PFS) was defined as the time from diagnosis to the date of any relapse or death. Overall survival (OS) was defined as the time from diagnosis until death from any cause or last follow-up. DPFS, PFS, and OS were performed with the Kaplan–Meier method. Cox proportional hazards analysis was performed to investigate the impact of age, PSA level (at diagnosis, postoperative, and at relapse), GS, hormone therapy, chemotherapy, adjuvant radiotherapy, and relapse site on PFS, DFS, and OS of the whole cohort, on the PET-positive and PET-negative subgroups; PFS and OS were then evaluated in the population stratified by risk category and PSA at diagnosis. The analyses were assessed with Stata software, version 16.0, and a 5% significance level was applied to all the tests.

## Results

### Population characteristics

The patients’ characteristics are summarized in **Table 1**. The median PSA value at diagnosis was 10 ng/ml (IQR 7-20 ng/ml). Twenty-nine (7.1%), 166 (40.5%), and 215 (52.4%) patients were at low, intermediate, and high risk, respectively. All patients underwent prostatectomy, and the median postoperative PSA was 0.02 ng/dl (IQR undetectable–0.05). Ninety-two (22.4%) patients received ADT, and adjuvant radiotherapy to the prostatic bed was performed in 98 patients (23.9%). Mean PSA at BR was 1.23 (IQR 0.63-2.45). At the time of BR, patients underwent restaging with choline PET/CT, and subsequent therapeutic strategies were adopted: EBRT to the prostate bed, ADT, chemotherapy, or stereotactic body radiotherapy (SBRT) either to the pelvic LNs or to oligometastases. At relapse, 215 patients (52.8%) received ADT and 27 patients (6.6%) underwent chemotherapy. Among the 410 patients analyzed, 176 (42.9%) patients had a negative ^18^F-choline PET/CT, while 234 (57.1%) patients had a positive PET/CT. This latter cohort included 223 (54.4%) patients with one to three areas of high uptake and 11 (2.7%) with more than three positive sites. Patients with a negative PET/CT had a higher GS at diagnosis and lower postoperative and at recurrence PSA values compared with the positive ^18^F-choline PET/CT scan cohort. We compared the subset of patients with positive ^18^F-choline PET/CT treated with SBRT (*n* = 73) to those who underwent other treatments (*n* = 161). Baseline and post-surgery PSA, risk classes, adjuvant treatments, and other characteristics of the two groups of patients are described in **Table 2**. The median PSA at recurrence in the two subgroups was 1.22 ng/dl (IQR 0.69-2.43) and 1.69 ng/dl (IQR 1.00-3.81), respectively. At recurrence, 34 (47.2%) and 91 (57.2%) received ADT, while 1 (1.4%) and 11 (6.9%) had chemotherapy, respectively. ^18^F-choline PET/CT-positive sites ranged from one to three in 150 (93.2%) patients and more than three in 11 (6.8%) patients. All patients treated with SBRT had one to three positive sites. In two groups (patients treated with SBRT *vs*. those not treated with SBRT), the PET/CT-positive sites were as follows: pelvic (26.9% *vs*. 63%) and lumbar LNs (3.8% *vs*. 5.5%), prostate (60.6% *vs*. 17.8%), bone (20.6% *vs*. 21.9%), and lung (0.6% *vs*. 0).

**Table 1:** Patients’ main characteristics.

	Total study sample (*n* = 410)	
	*N* or median	% or IQR
Age at diagnosis	66	(62-70)
PSA at diagnosis	10	(7-20)
Gleason at diagnosis		
≤6 (low risk)	29	7.1%
4+3 (intermediate risk, favorable)	71	17.3%
3+4 (intermediate risk, unfavorable)	95	23.2%
≥8 (high risk)	215	52.4%
Hormone therapy (initial)		
No	318	77.6%
Yes	92	22.4%
PSA post-surgery	0.02	(0.0-0.5)
Adjuvant radiotherapy		
No	312	76.1%
Yes	98	23.9%
PSA at recurrence	1.23	(0.63-2.45)
Recurrence (% yes)		
Lumbar nodes	10	2.4%
Pelvic nodes	89	21.7%
Bone	49	12.0%
Lung	1	0.2%
Prostate	110	26.8%
No. of positive sites at PET		
0	176	42.9%
1-3	223	54.4%
>3	11	2.7%
Hormone therapy (at recurrence)		
No	192	47.2%
Yes	215	52.8%
Chemotherapy (at recurrence)		
No	380	93.4%
Yes	27	6.6%

**Table 2:** Impact of clinical patients and disease characteristics on PET Choline results.

	PET-positive (n=161) N or median % or IQR		PET-positive + SBRT (n=73) N or median % or IQR		p-value
**Age at diagnosis**	66	(62-69)	66	(62-70)	0,153
**PSA at diagnosis**	10	(6.7-17.7)	9,8	(7-19)	0,920
**Gleason at diagnosis**					
≤6 (low risk)	13	8,1%	4	5,5%	
4+3 (intermediate risk, favourable)	26	16,1%	13	17,8%	
3+4 (intermediate risk, unfavourable)	52	32,3%	19	26,0%	
≥8 (high risk)	70	43,5%	37	50,7%	0,133
**Hormone therapy (initial)**					
no	131	81,4%	58	79,5%	
yes	30	18,6%	15	20,5%	0,210
**PSA post-surgery**	0,6	(0.0-0.71)	0,02	(0.0-0.5)	0,123
**Adjuvant radiotherapy**					
no	133	82,6%	44	60,3%	
yes	28	17,4%	29	39,7%	0,001*
**PSA at recurrence**	1,69	(1.00-3.81)	1,22	(0.69-2.43)	<0.001*
**Recurrence (% yes)**					
lumbar nodes	6	3,8%	4	5,5%	0,015*
pelvic nodes	43	26,9%	46	63,0%	<0.001*
bone	33	20,6%	16	21,9%	<0.001*
lung	1	0,6%	0	0,0%	0,461
prostate	97	60,6%	13	17,8%	<0.001*
**N positive sites at PET**					
0	0	0,0%	0	0,0%	
1-3	150	93,2%	73	100,0%	
>3	11	6,8%	0	0,0%	<0.001*
**Hormone therapy (at recurrence)**					
no	68	42,8%	38	52,8%	
yes	91	57,2%	34	47,2%	0,309
**Chemotherapy (at recurrence)**					
no	148	93,1%	71	98,6%	
yes	11	6,9%	1	1,4%	0,120

(*) significant results.

### Clinical outcomes

**Figure 1:**
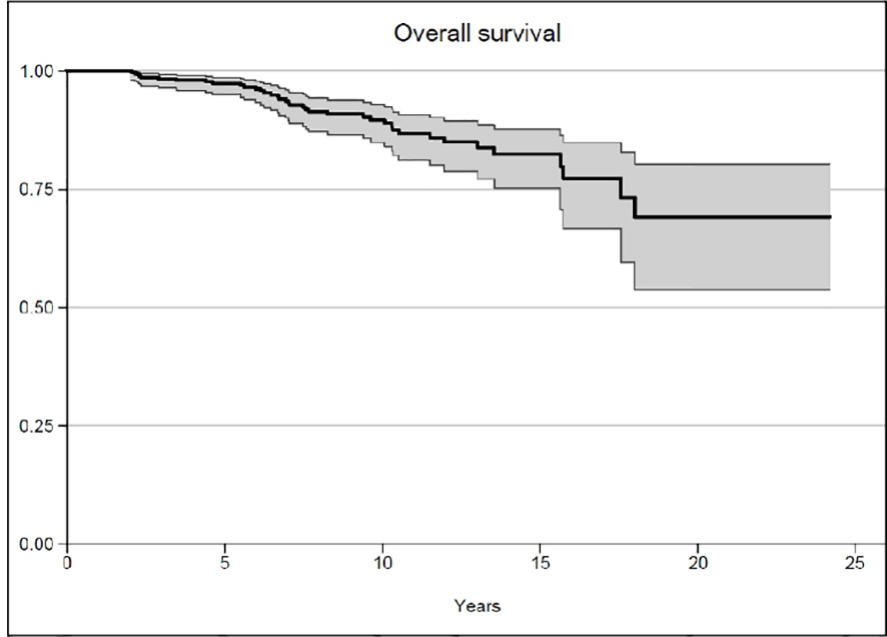
Overall survival.

**Figure 2:**
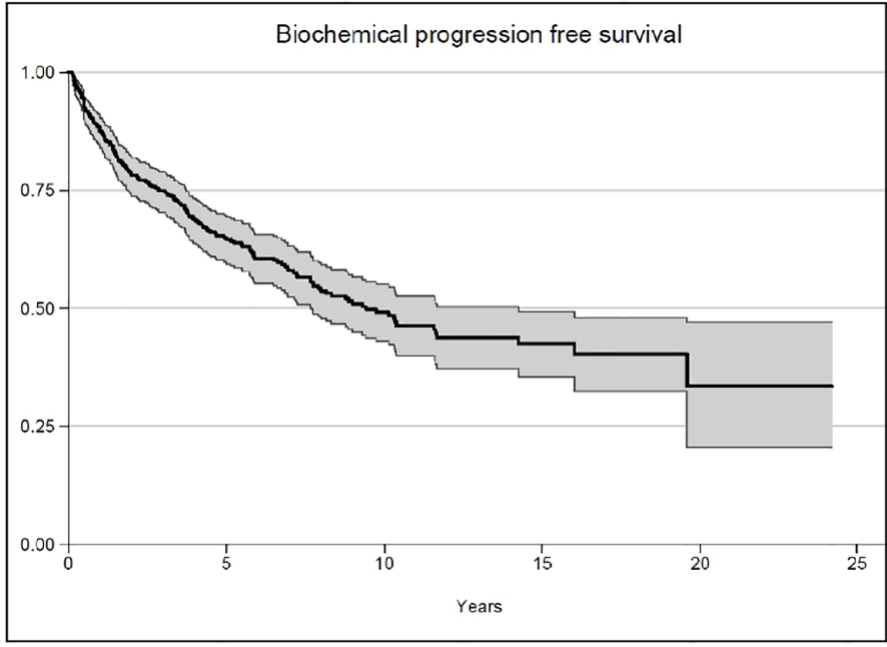
Biochemical progression-free survival.

**Figure 3:**
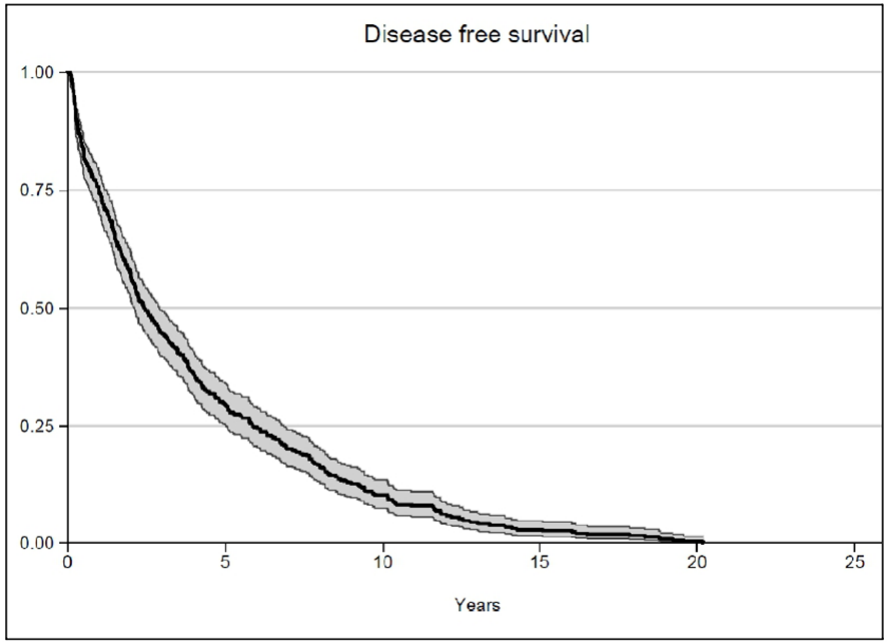
Disease-free survival.

After a median follow-up of 7.9 years, the median OS was not reached, while 93.4% of the patients survived 5 years and 89.6% survived 10 years (**Figure 1**). The median biochemical PFS was 9.4 years, with 64.9% of the patients stable at 5 years and 49.1% of the patients without biochemical relapse at 10 years (**Figure 2**). The median DFS was 2.4 years, with 29.6% stable patients at 5 years and 10.3% stable patients at 10 years (**Figure 3**). Moreover, we did not report any statistical difference in terms of OS and PFS between the PET-negative and the PET-positive subsets.

### Prognostic features

In the univariate analysis, post-surgery and recurrence PSA, chemotherapy, and the number of metastasis sites significantly impacted the OS in all cohorts (**Table 3**); in the multivariate analysis, only chemotherapy and PSA at recurrence represented significant factors. In the multivariate analysis, the number of sites of relapses and chemotherapy had an impact on OS in the PET-positive subgroup, while only chemotherapy had an impact on OS in the PET-negative subgroup (**Table 3**). In the univariate analysis, PSA at recurrence and GS at diagnosis had an impact on PFS in all populations (**Table 4**), while in the PET-positive and PET-negative subgroups, post-surgery and PSA at recurrence had an impact on PFS in the univariate analysis (**Table 4**). Finally, in the univariate analysis, the DFS depended on GS at diagnosis, post-surgery, and recurrence PSA in the whole population, but only post-surgery PSA and GS were confirmed as prognostic factors in the multivariate analysis (**Table 5**). In the multivariate analysis, post-surgery PSA, GS, and the number of sites had an impact on the DFS of patients with positive PET images (**Table 5**), while in the PET-negative subgroup, antiandrogenic therapy, post-surgery, and at recurrence PSA had an impact on the DFS (**Table 5**).

**Table 3:** Results of the univariate and multivariate analyses of OS in the total cohort and in the PET-positive and PET-negative subgroups.

Criteria	All populations		PET-positive subgroup	PET-negative subgroup
	Univariate analysis	Multivariate analysis	Multivariate analysis	Multivariate analysis
	*P*-value HR (95% CI)	*P*-value HR (95% CI)	*P*-value HR (95% CI)	*P*-value HR (95% CI)
Recurrence PSA (≥1.2 *vs*. <1.2)	0.009* 2.86 (1.30-6.27)	0.027* 2.53 (1.11-5.75)		0.055 2.57 (0.98-6.76)
Post-surgery PSA (≥0.2 *vs*. <0.2)	0.044* 1.95 (1.02-3.75)		0.051 3.42 (0.99-11.79)	
No. of positive sites at PET/TC 1-3 >3	0.037* 0.47 (0.23-0.96) 0.031* 3.82 (1.13-12.91)		0.001* 9.17 (2.40-35.04)	
Chemotherapy at recurrence (yes *vs*. no)	0.009* 2.86 (1.30-6.27)	0.001* 3.95 (1.81-8.64)	0.043* 4.24 (1.05-17.18)	0.006 3.81 (1.47-9.90)
Hormone therapy at recurrence (yes *vs*. no)	0.081 1.87 (0.93-3.78)			

*Significant results.

**Table 4:** Results of the univariate analysis of PFS in the total cohort and in the PET-positive and PET-negative subgroups.

Criteria	All populations	PET-positive subgroup	PET-negative subgroup
	*P*-value HR (95% CI)	*P*-value HR (95% CI)	*P*-value HR (95% CI)
Recurrence PSA (≥1.2 *vs*.< 1.2)	<0.001* 0.49 (0.36-0.67)	0.012* 0.64 (0.45-0.91)	0.030* 0.71 (0.52-0.97)
Post-surgery PSA (≥0.2 *vs*. <0.2)		0.009* 1.79 (1.16-2.76)	0.088* 1.32 (0.96-1.81)
GS at diagnosis (≥8 *vs*. 4 + 3) (≥8 *vs*. 3 + 4) (≥8 *vs*. <6)	0.520 0.88 (0.59-1.30) <0.001* 0.35 (0.22-0.54) 0.060* 0.55 (0.30-1.02)		

*Significant results.

**Table 5:** Results of the univariate and multivariate analyses of DFS in the total cohort and in the PET-positive and PET-negative subgroups.

Criteria	All populations		PET-positive subgroup	PET-negative subgroup
	Univariate analysis	Multivariate analysis	Multivariate analysis	Multivariate analysis
	*P*-value HR (95% CI)	*P*-value HR (95% CI)	*P*-value HR (95% CI)	*P*-value HR (95% CI)
Recurrence PSA (≥1.2 *vs*.<1.2)	<0.001* 1.84 81.50-2.25)			0.012 0.64 (0.45-0.91)
Post-surgery PSA (≥0.2 *vs*.0.2)	0.039* 0.81 (0.67-0.99)	<0.001* 2.47 (1.88-3.25)	<0.001* 3.62 (2.48-5.30)	0.007 1.84 (1.118-2.86)
Chemotherapy at recurrence (yes *vs*. no)	0.562 1.12 (0.76-1.66)			
Hormone therapy at recurrence (yes *vs*. no)	0.091 1.18 (0.97-1.44)			0.033 1.48 (1.03-2.13)
GS at diagnosis (≥8 *vs*. 4 + 3) (≥8 *vs*. 3 + 4) (≥8 *vs*. <6)	0.338 0.88 (0.67-1.15) <0.001* 0.59(0.46-0.75) <0.01* 0.48(0.32-0.71)	0.334 0.87 (0.65-1.16) 0.001* 0.63 (0.48-0.83) <0.001* 0.41 (0.26-0.63)	0.613 0.90 (0.59-1.36) 0.012* 0.64 (0.46-0.91) <0.001* 0.30 (0.16-0.55)	
No. of positive sites at PET/TC (>3 *vs*. 1-3)			0.099* 1.70 (0.90-3.21)	

*Significant results.

Among patients with low/intermediate risk according to GS, factors that were independently associated with worse OS were age (≥65 years), post-surgery PSA (≥0.02), and the number of PET-positive sites (>3). Among high-risk patients, the number of PET-positive sites and chemotherapy at recurrence were associated with worse OS. After stratifying by PSA at diagnosis, factors associated with poor OS were the number of PET-positive sites (>=3) among patients with PSA at diagnosis above 10 and the use of chemotherapy at recurrence in patients with PSA at diagnosis both below and above 10 (although the association was stronger in the former group). In terms of PFS, a higher PSA at recurrence (>=1.2) was the only individual factor associated with a longer survival among patients with low/intermediate and high risk according to GS. Among patients with PSA at diagnosis below 10, no factor significantly affected PSA, while among those with PSA at diagnosis above 10, a worse PFS was associated with lower PSA (post-surgery and at recurrence) and with having been treated with chemotherapy at recurrence.

## Discussion

This retrospective study strengthens our experience and the recent literature on choline PET/CT for patients with post-surgical BR. We analyzed a large cohort who had undergone restaging with ^18^F-choline PET/CT at BR. Moreover, we highlighted the value of choline PET/CT in the early diagnosis of macroscopic recurrence, thus impacting subsequent treatment.

Both univariate and multivariate analyses confirmed the effect of chemotherapy as the first therapeutic choice and low PSA at recurrence on OS, thus highlighting the role of choline PET/CT in accurate disease staging and in the diagnostic–therapeutic paradigm. Moreover, both post-surgery PSA and GS were prognostic factors for DFS in the multivariate analysis. It is legit to infer that choline PET/CT allows the early diagnosis of relapse in patients with BR, to distinguish different patient settings and to choose the most suitable management.

The impact of choline PET/CT is to be able to identify the best therapeutic treatment for patients with post-surgery BR with low PSA value. By providing more accurate restaging either in the local, nodal, or metastatic setting, choline PET/CT can potentially lead to significant adjustments in treatment management in the postoperative setting, ranging from 35% to 64% (11). PET/CT distinguishes between PET-positive (macroscopic recurrence) and PET-negative (microscopic recurrence) subsets. In the PET-positive subset, there are different patient settings for the number and site of relapses (prostate bed, locoregional drainage lymph nodes, and distant metastases), and each different setting needs a distinct therapeutic management. Within the known limits of sensitivity of choline PET/CT, the choice of the most appropriate therapy is mainly guided by the radiological burden of disease. Indeed, a limited burden of disease can benefit from more targeted treatments (SBRT, for example, is the treatment of choice when the PET-positive sites are between 1 and 3). This therapeutic strategy allows to maintain disease control without necessarily resorting to systemic therapies, but the response monitoring for such treatment currently depends on PSA serum level and CT scans.

In the clinical scenario of PCa with BR, we had consistent early experience in our center with ^18^F-choline PET/CT, reporting it as a useful diagnostic tool for detecting early relapse in patients with rising PSA after primary treatment with the notable limitation of the need to have PSA values higher than 1 ng/dl (12).

There is a growing body of literature showing increased sensitivity and specificity for next-generation imaging techniques. A recently developed novel radiotracer targeting prostate-specific membrane antigen (PSMA) has shown potential in this field, particularly in cases of low PSA value at recurrence (13, 14). In cases of BR after prostatectomy, the most recent guidelines of the European Association of Urology recommend PSMA PET, although with limited strength of recommendation and only in limited cases where results will influence subsequent treatment decisions (10). Our data showed that at a median recurrence PSA value <1 ng/dl the risk of choline-negative PET/CT scan increased, thus confirming the higher sensitivity of PSMA PET in relapsed prostate cancer. However, it should be acknowledged that most of the PSMA PET data are based on retrospective series, and the high cost and limited availability of this method are still major limitations (15). Therefore, taking into account also the heterogeneity of the disease and that almost 10% of PCa is PSMA negative, we believe that choline PET/CT still represents an effective molecular imaging technique that should be considered (16).

The PSA level at the time of choline PET/CT imaging was the most crucial parameter to predict scan positivity: higher GS, higher PSA velocity, persistently elevated PSA after initial treatment, and initial treatment with RT increased the probability for positive choline scans (17). The use of new prostate-specific tracers providing superior spatial and temporal resolution compared with commercially available PET scanners will undoubtedly play increasing roles in defining the presence and extent of relapsing disease and will promote the development and use of precision therapies in patients with relapsing prostate cancer. Wang et al. (18) recently published a meta-analysis to compare the diagnostic role of ^18^F-choline, ^18^F-fluciclovine, and ^18^F-PSMA PET/CT in the detection of PCa with BR: the sensitivity and specificity of ^18^F-choline and ^18^F-fluciclovine PET/CT were 0.93 and 0.91 and 0.80 and 0.66, respectively. The pooled detection rates of ^18^F-choline, fluciclovine, and PSMA were 66%, 74%, and 83%, respectively. The study revealed that ^18^F-PSMA had the highest detection rate at different PSA levels and also for patients with biochemical recurrence after radical prostatectomy with low PSA concentrations (≤2.0 ng/ml).

Our experience with PSMA PET has confirmed its detection rate to be of excellent accuracy and reliability: it can influence the clinical management of a relevant percentage of patients, confirming the chance of personalized treatment and an overall better disease control with the subsequent treatment we selected using SBRT alone, concomitant SBRT and ADT, or salvage prostate bed RT (19).

The role that CT may have in customizing the therapeutic algorithm and in improving clinical outcomes has long been questioned. Changes in management based on molecular PET imaging result in the escalation of treatment: modifications of volume and dose, intensification of radiotherapy, or de-intensification of concurrent ADT (20). The PET radiotracers allow the early diagnosis of recurrence in PCa, and PET images can guide local therapies in patients with limited disease relapse (21) by postponing systemic treatments.

Several retrospective experiences have shown that by treating with local ablative approaches, such as SBRT, approximately 40% of patients were biochemical recurrence-free and 71% were clinical disease-free, with very low rates of toxicities (between 0% and 5%) (22). Similarly, a prospective trial screened patients with biochemical recurrence after first-line curative treatments; the patients were restaged using ^18^F-FMCH-PET/CT, and salvage treatment was selected based on the imaging findings. Patients with <4 synchronous metastases were treated with repeated SBRT on all the detected lesions. The trial showed that the median systemic therapy-free survival was 39.1 months, while the systemic-free survival ratio at 24 months was 63.1%; finally, the decline between baseline SA pre-SBRT and PSA value 6 weeks after treatment (delta PSA) demonstrated an impact on systemic therapy-free survival (23). In this clinical scenario, we present the results of a multicenter, randomized trial (ARTO-NCT03449719): patients with oligometastatic (with less than three non-visceral metastatic lesions) castrate-resistant prostate cancer (CRPC) were randomized to receive either abiraterone acetate alone or associated with SBRT on all sites of disease. The preliminary results corroborate the efficacy and safety of SBRT (24). However, limited studies exist that report changes in outcome based on molecular PET imaging, while several randomized studies are currently underway.

Our study has some limitations: the inherently retrospective nature of our study and the limited follow‐up. Furthermore, we used ^18^F-choline PET/CT to assess patients with BR; new prostate-specific tracers are emerging with higher sensitivity and specificity, providing early detection and accurate definition of the extent of the disease at lower PSA values.

Although PSMA PET is more accurate and decidedly more promising in terms of results, the centers that guarantee the use of choline PET/CT are much more widespread, and the number of studies involving it makes the data concerning it considerably more solid.

In light of the increasing availability of PSMA PET, it is expected to become the imaging of choice in the evaluation of recurrent PCa.

## Conclusions

Choline PET/CT imaging is of paramount importance when it comes to restaging BC PCa after RP, proving better accuracy than conventional imaging and leading to changes in the management and intensification of treatment strategies. Ongoing studies will define the role of PSMA PET for salvage radiotherapy treatments after RP and prompt new perspectives in this unmet scenario.

## Data availability statement

The raw data supporting the conclusions of this article will be made available by the authors, without undue reservation.

## Ethics statement

The studies involving human participants were reviewed and approved by Area Vasta Toscana Centro. The patients/participants provided their written informed consent to participate in this study.

## Author contributions

DB, LL, CA, and GI were responsible for the conception of the study. LB, DS, and SF were responsible for the data collection. SC performed the data analysis. SS, GF, and VC interpreted the data. SL and MR drafted the manuscript. SS participated in the conception of the study and critically revised the manuscript for important intellectual content. All authors contributed to the article and approved the submitted version.
